# Clinical Outcomes Following Suture Fixation of Intraocular Lenses at the University Eye Clinic Freiburg: A Retrospective Analysis

**DOI:** 10.3390/jcm14072271

**Published:** 2025-03-26

**Authors:** Mateusz Glegola, Michelle Dreesbach, Daniel Böhringer, Philip Maier, Thomas Reinhard

**Affiliations:** Department of ophthalmology, University Clinic Freiburg, 79106 Freiburg, Germany; michelle.dreesbach@uniklinik-freiburg.de (M.D.); philip.maier@uniklinik-freiburg.de (P.M.); thomas.reinhard@uniklinik-freiburg.de (T.R.)

**Keywords:** IOL suture fixation, risk factors, refractive outcomes

## Abstract

**Background**: Suture fixation of intraocular lenses (IOLs) is a rare but essential procedure for patients with inadequate capsular support, offering crucial therapeutic benefits. This study analyzes a large cohort of patients undergoing IOL suture fixation, focusing on demographics, risk factors, and clinical outcomes. **Methods**: In this retrospective analysis of 332 eyes treated at the Eye Center of the University Hospital Freiburg (2008–2022), we evaluated immediate and long-term postoperative outcomes, including visual acuity and refraction. The follow-up averaged 2.5 years. A detailed analysis of the IOL positioning was performed in 111 patients. **Results**: The most common indications were pseudoexfoliation syndrome (33%), trauma (17%), and prior complicated cataract surgery (11%). The Zeiss CT27SF lens was used most frequently (91%). The mean spherical equivalent deviation from target refraction was −0.375 diopters overall. According to the detailed analysis, IOL tilt occurred in 6.3% of patients, and decentration occurred in 7.2%. Only three patients (3.6%) required additional ocular procedures specifically for IOL repositioning. Risk factors for further surgery included underlying systemic conditions or multiple previous surgeries. **Conclusions**: Suture fixation of IOLs proves to be an adequate and effective intervention for visual rehabilitation in patients with compromised capsular support, demonstrating good refractive outcomes and low complication rates. Even in cases where the postoperative visual acuity was comparable to the best-corrected preoperative acuity, the procedure improved refractive correction by reducing the need for extensive refractive aids, such as contact lenses required for aphakia.

## 1. Introduction

Intraocular lens (IOL) implantation has become the standard of care in modern cataract surgery, with most IOLs being safely placed within the capsular bag. However, in certain clinical scenarios, conventional in-the-bag IOL implantation may not be possible due to insufficient capsular support. This presents a significant challenge in both primary and secondary IOL implantation procedures.

**Figure 1:**
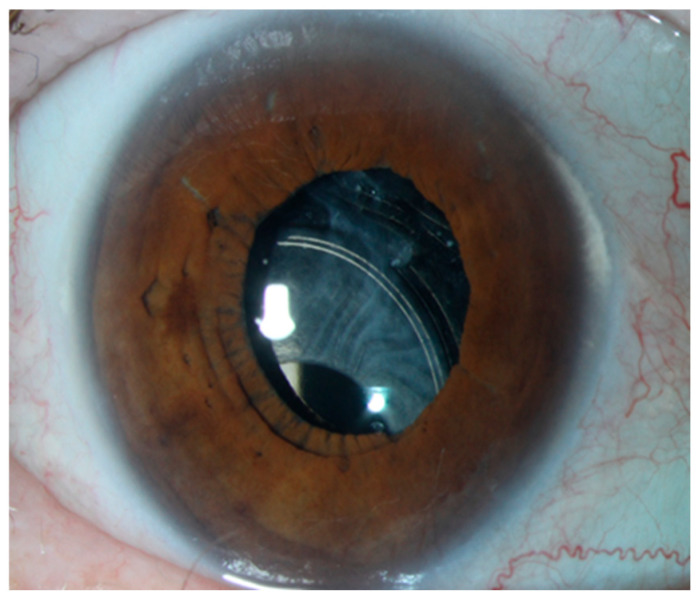
IOL dislocation in a patient with pseudoexfoliation syndrome.

Various conditions can compromise capsular integrity, including pseudoexfoliation syndrome (PEX) (see Figure 1), trauma, complicated previous cataract surgery, and congenital disorders [1,2]. In such cases, alternative IOL fixation techniques become necessary. The main surgical options include anterior chamber IOLs, iris-fixated IOLs (both posterior and anterior chamber lenses), and posterior chamber IOLs with scleral or sutured fixation [3,4].

The surgical approaches for IOL fixation have evolved significantly over in recent decades. Anterior chamber IOLs, while technically less demanding, carry risks of endothelial cell loss, peripheral anterior synechiae, and secondary glaucoma. Iris-fixated IOLs can provide good centration but may lead to iris chafing, pigment dispersion, and chronic inflammation [1]. Techniques for posterior chamber IOL fixation include transscleral suture fixation and sutureless scleral fixation (Yamane technique, glued IOL techniques, and intrascleral haptic fixation) [5,6,7,8,9,10,11].

Each technique has specific advantages and limitations. Suture fixation of posterior chamber IOLs, while technically more challenging, offers several benefits: it positions the IOL closer to the physiological lens position, provides stable fixation, and allows for precise IOL centration. This optimal positioning typically results in better optical outcomes and reduced higher-order aberrations compared to anterior chamber IOLs. The posterior placement also minimizes contact with anterior segment structures, thereby reducing the risk of endothelial cell loss, a common concern with anterior chamber IOLs. Additionally, sutured posterior chamber IOLs show excellent long-term stability when proper fixation techniques are employed, and, unlike iris-fixated lenses, they do not depend on iris integrity or stability. This makes them particularly suitable for trauma cases or patients with iris abnormalities. The technique also offers greater flexibility in IOL power selection and positioning adjustments during surgery, allowing for optimal refractive outcomes. The suture-fixated technique has been further refined to address early complications, such as suture erosion and IOL tilt. Different suture types can be used (polypropylene, Gore-Tex, and Z-suture) [9,10,12,13,14,15]. While newer sutureless techniques like the Yamane method have gained popularity, sutured fixation remains particularly valuable in cases with complex anatomy, in revision surgeries, or when maximum stability is required.

Despite its clinical importance, long-term data on outcomes following IOL suture fixation remain relatively limited [16].

Previous studies have typically involved smaller cohorts or shorter follow-up periods, leaving questions about long-term stability and complication rates. Furthermore, the relationship between different underlying pathologies and refractive and surgical outcomes has not been extensively studied.

In our study, we aim to evaluate the long-term clinical outcomes and safety profile of sutured IOLs, identify risk factors and their impact on surgical success, assess the refractive outcomes with different IOL types, and analyze the rate and nature of postoperative complications.

This extensive analysis will provide valuable insights into the efficacy and safety of IOL suture fixation, helping to guide surgical decision-making and improve patient outcomes.

## 2. Materials and Methods

### 2.1. Study Design and Patient Selection

This retrospective study analyzed all cases of IOL suture fixation performed at the Eye Center of the University Hospital Freiburg between 2008 and 2022. The study adhered to the tenets of the Declaration of Helsinki and was approved by the local ethics committee. Inclusion criteria required the availability of both pre- and postoperative data and at least one follow-up visit at our institution. Cases were identified through the hospital’s surgical database, and medical records were reviewed for relevant data.

### 2.2. Outcome Measures

Primary outcome measures included visual acuity (BCVA), refractive outcomes (spherical equivalent deviation from target), IOL position stability (tilt and decentration), and complication rates requiring further surgery.

Secondary outcomes included the correlation between underlying pathologies and surgical outcomes and long-term IOL stability. A detailed analysis of IOL positioning was performed in a subgroup of 111 randomly selected patients.

### 2.3. Data Collection

Collected data included patient demographics, preoperative factors and indications for surgery, type of IOL used, preoperative and postoperative visual acuity, refractive outcomes, surgical complications, and duration of follow-up.

### 2.4. Surgical Technique

All surgeries followed a standardized technique involving transscleral fixation of posterior chamber IOLs. The procedure included the creation of conjunctival peritomies at 3 and 9 o’clock, episcleral cauterization and the creation of triangular lamellar scleral flaps, placement of single 10–0 nylon sutures deep in each flap, ab externo passage of 10–0 Prolene sutures using scleral needles, IOL haptic fixation and positioning in the ciliary sulcus, tying of prolene and nylon sutures and burial under scleral flaps, and conjunctival closure. In all cases, the capsular bag was removed prior to IOL fixation to avoid potential long-term complications associated with residual capsular material. The IOL was therefore always sutured without the bag. Additionally, a vitrectomy was performed to optimize IOL stability and minimize vitreous traction. Depending on intraoperative requirements, either a pars plana vitrectomy or an anterior vitrectomy was conducted.

### 2.5. Statistical Analysis

All analyses were performed using R statistical software (version 4.2.3). For demographic and baseline characteristics, descriptive statistics were calculated with means, medians, and ranges for continuous variables and with frequencies and percentages for categorical variables.

Visual outcomes were analyzed in two ways: First, raw visual acuity values were summarized using medians and ranges. Second, to visualize patterns of visual change, we created a Sankey diagram by categorizing visual acuity into four groups (≤0.05, >0.05–0.20, >0.20–0.50, and >0.50–1.60) and tracking transitions between preoperative and postoperative states.

**Figure 2:**
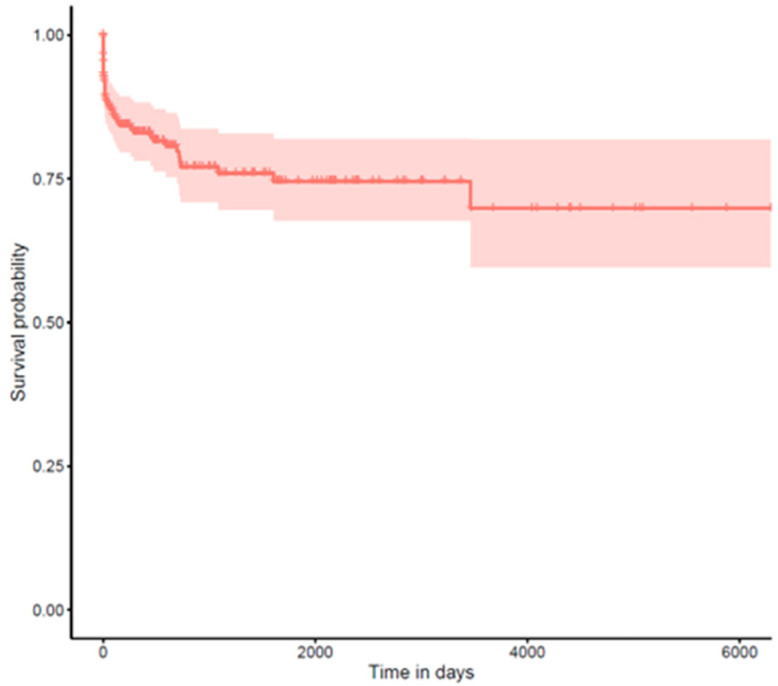
Kaplan–Meier curve for long-term complications: glaucoma.

**Figure 3:**
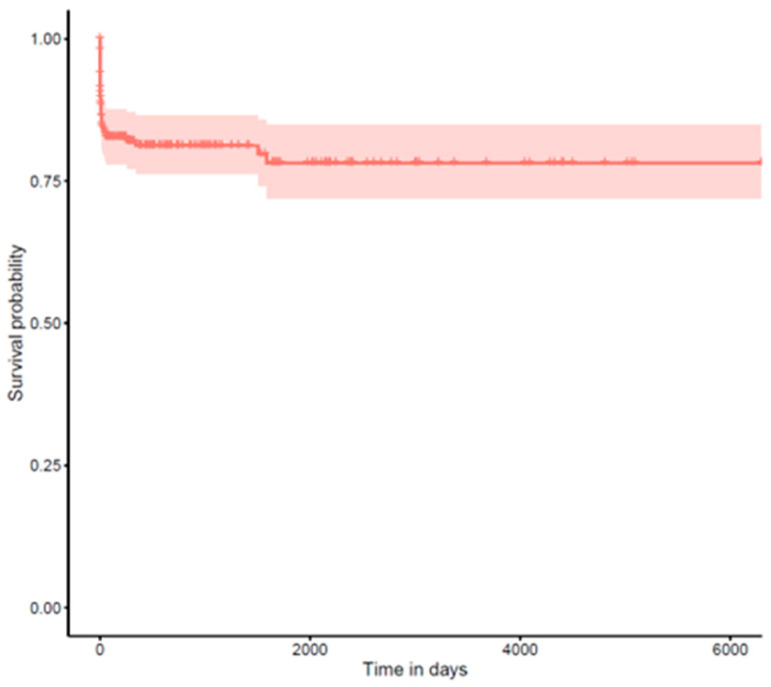
Kaplan–Meier curve for long-term complications: retinal detachment.

For long-term complications (glaucoma and retinal detachment), we performed time-to-event analyses using the Kaplan–Meier method (see Figure 2 and Figure 3). Time was calculated from the date of surgery until either the occurrence of the complication or the last documented follow-up visit (censoring). Median survival times with 95% confidence intervals were calculated where the event rate allowed.

IOL position outcomes were analyzed in a randomly selected subgroup of 111 patients using descriptive statistics. Decentration and tilt were categorized as none, mild, or severe based on standardized clinical assessment.

Refractive outcomes were summarized using means and ranges of spherical equivalent deviation from target refraction, calculated separately for each IOL type. Missing data were handled using complete case analysis for the respective outcomes, with no imputation performed. The number of available cases is reported for each analysis.

## 3. Results

Of all IOL suture fixation procedures performed at the University Eye Clinic Freiburg between 2008 and 2022, 332 eyes that underwent IOL suture fixation and had at least one follow-up visit at our institution were included in this analysis. The mean follow-up period was 2.5 years, with a median of 0.8 years (range: 1 day to 17.23 years). Fifty-two cases were excluded from survival analyses due to missing follow-up data.

### 3.1. Risk Factors and Indications

**Figure 4:**
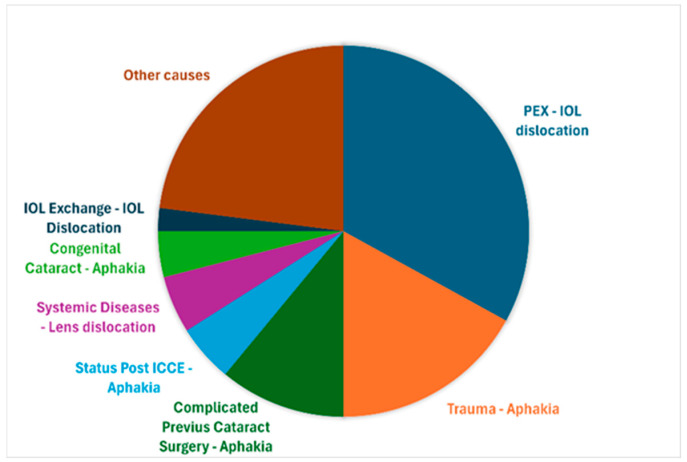
Indications for suture-fixated intraocular lenses.

The most common indications for IOL suture fixation were IOL dislocation due to pseudoexfoliation syndrome (33%), aphakia following trauma (17%), and aphakia following complicated previous cataract surgery (11%). Other indications included aphakia after intracapsular cataract extraction (ICCE) (5%), lens dislocation due to systemic diseases (e.g., Marfan syndrome or homocystinuria) (5%), aphakia after congenital cataract extraction (4%), and IOL exchange due to IOL opacification (2%). Various other causes (23%) included IOL dislocation due to chronic inflammatory conditions such as uveitis and status after multiple surgeries (e.g., vitrectomy for retinal detachment) (see Figure 4).

### 3.2. IOL Types and Refractive Outcomes

The Carl Zeiss Meditec AG CT27SF Lens, a monobloc IOL, was the most frequently used IOL type (92%, *n* = 303), followed by the Alcon Deutschland GmbH MA60AC Lens, a three-piece IOL (3%, *n* = 11), and other IOL models (5%, *n* = 18). The mean spherical equivalent deviation from target refraction was −0.375 diopters. Specifically, for CT27SF, the mean deviation was 0.3 D (range: −0.86 to 1.69 D), that for MA60AC was 0.0 D (range: −0.06 to 1.1 D), and that for other IOLs was 0.5 D (range: −0.1 to 1.12 D) (see Table 1).

### 3.3. Visual Acuity Outcomes

**Figure 5:**
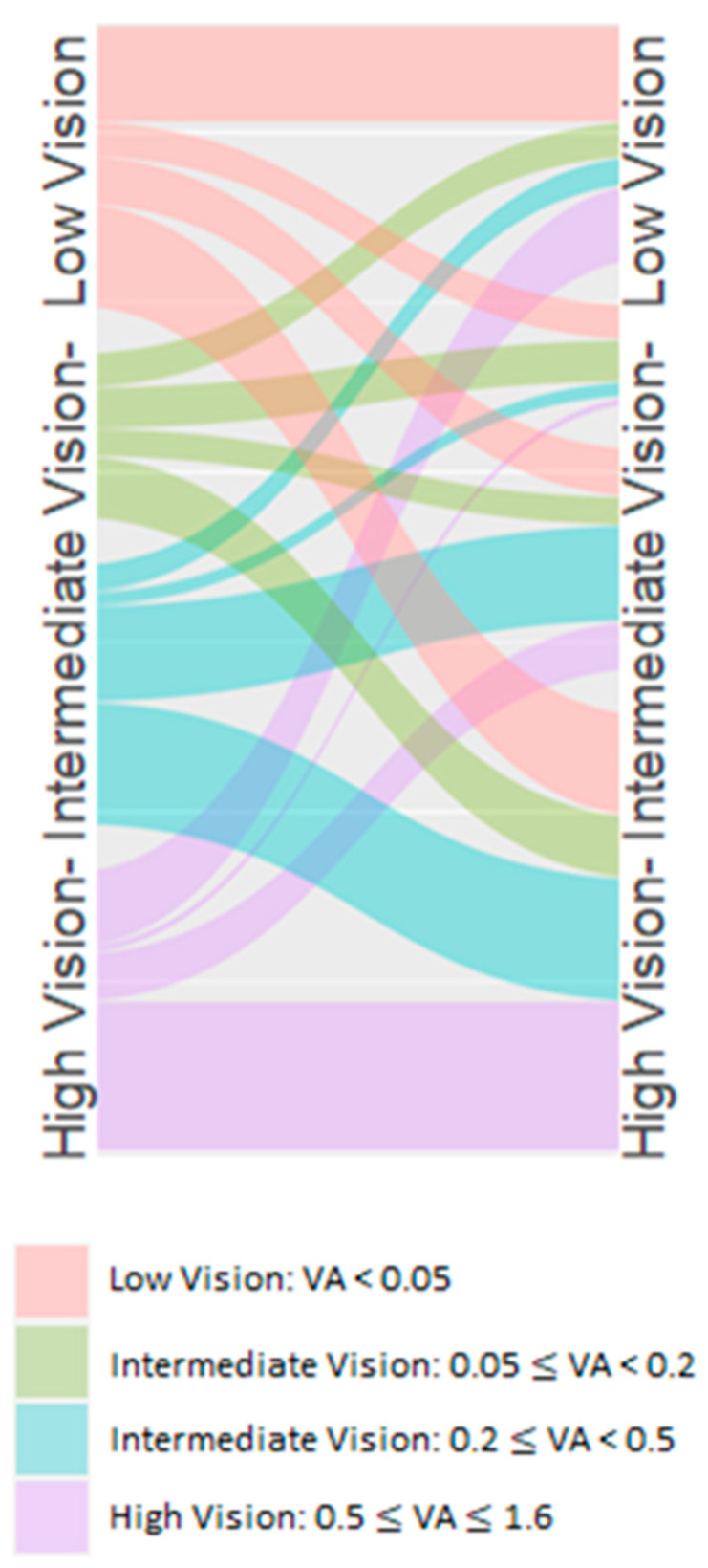
Visual transitions from pre- to postoperative states.

The visual transitions from pre- to postoperative states reveal an encouraging pattern of visual rehabilitation following IOL suture fixation. Approximately two-thirds of the patients included in this study experienced meaningful visual improvement, with many achieving moderate or even good vision postoperatively. This upward trend in visual function was particularly noteworthy given the complexity of these cases. However, every third patient who entered surgery with good vision (0.20–0.50) lost visual function at the end of the follow up to some degree (see Figure 5).

The overall pattern suggests that IOL suture fixation stabilizes vision in most cases but offers potential for visual improvement if the preoperative visual acuity is low. In cases where the postoperative visual acuity did not show significant improvement compared to the best-corrected preoperative level, patients still benefited from a more favorable refractive situation with a reduced need for intensive refractive correction, particularly contact lenses for aphakic correction.

### 3.4. IOL Position

In the detailed IOL positioning subgroup (*n* = 111), the position was favorable in the majority of cases: 93.7% showed no tilt (*n* = 104) and 92.8% (*n* = 103) were well centered. Among the cases with complications, 6.3% showed IOL tilt (4.5% severe, 2.7% mild), 7.2% showed decentration, and 3.6% required further surgery for the IOL, primarily patients with underlying systemic conditions (e.g., Marfan syndrome) or multiple previous surgeries.

### 3.5. Long-Term Complications

We conducted survival analyses for two major long-term complications in 280 patients (52 cases had insufficient follow-up data for inclusion). The median follow-up time for these analyses was 2.5 years, with some patients followed up with for up to 17 years.

### 3.6. Secondary Glaucoma

During the follow-up period, 47 patients (16.8%) developed secondary glaucoma. The cumulative incidence remained relatively low in the early postoperative period, with most cases developing after the first year.

### 3.7. Retinal Detachment

Retinal detachment occurred in 45 patients (16.1%). The survival curve showed a relatively steady occurrence rate throughout the follow-up period, suggesting that vigilance for this complication should be maintained during long-term follow-up.

## 4. Discussion

This large retrospective analysis of 332 eyes undergoing IOL suture fixation provides important insights into the long-term outcomes of this challenging surgical procedure. Our findings demonstrate that suture fixation is an adequate and effective approach across a wide spectrum of indications, particularly given the extended follow-up period of up to 17 years. However, it is important to acknowledge certain limitations, including the relatively shorter mean follow-up period of 2.5 years compared to other studies, as shown in Table 2.

Despite this, the mean follow-up period of 2.5 years is still substantial and provides meaningful insights, particularly given the high number of included cases (332 eyes). In comparison, previous studies with longer follow-up periods often analyzed significantly fewer eyes. For instance, Kokame et al. reported a mean follow-up of 72 months but included only 118 eyes, while Dimopoulos et al. had a mean follow-up of 64 months with 66 eyes, and Wasiluk et al. reported 64 months with only 29 eyes (see Table 2).

The high number of cases in our study can be attributed to several factors. First, the University Eye Clinic Freiburg serves as a tertiary referral center, receiving a large number of complex cases from a vast area of surrounding regions. This makes our patient population particularly large, diverse, and representative of the challenges faced in managing eyes with insufficient capsular support. Second, the extended period of data collection (2008–2022) allowed for the accumulation of a substantial number of cases, providing a robust dataset for analysis. Lastly, the use of standardized surgical techniques and consistent postoperative management across all cases ensured a high level of uniformity in treatment. This approach not only enhanced the reliability of data collection but also minimized inter-surgeon variability, thereby strengthening the internal validity of our findings. As a result, our study provides a particularly robust dataset for evaluating long-term outcomes in a well-defined and systematically treated patient cohort. This underscores the strength of our study: the comprehensive data were derived from a large cohort, making it one of the more extensive datasets available on this topic. The large sample size enables more robust conclusions regarding the safety and efficacy of suture-fixated IOLs as well as detailed subgroup analyses.

While the relatively shorter mean follow-up could be viewed as a limitation, it is offset by the inclusion of long-term follow-up data for some patients (up to 17 years) and the sheer number of cases analyzed. Together, these factors make our dataset valuable for understanding both short- and mid-term outcomes and provide a strong foundation for further research into long-term trends.

The predominance of IOL dislocation due to pseudoexfoliation syndrome (33.1%) as the primary indication aligns with the previous literature [1,9,15,18] and underscores the importance of the early recognition of zonular weakness in these patients. The substantial proportion of trauma cases (17.5%) reflects both the tertiary nature of our center and the particular utility of suture fixation in managing complex traumatic cases where conventional IOL placement is not feasible.

The Sankey analysis of visual acuity transitions reveals that most patients maintained or improved their vision postoperatively, with particularly meaningful gains in those presenting with poor initial vision. In cases where the best-corrected preoperative visual acuity was similar to the postoperative level, refractive outcomes were still optimized by reducing the need for high refractive correction, offering a more manageable refractive solution without the dependence on contact lenses typically necessary for aphakia. This suggests that even in cases with limited visual potential, surgical intervention can offer significant benefit, also shown in the previous literature [10].

The refractive outcomes, with a mean spherical equivalent deviation of only −0.375 diopters, compare favorably with published results for standard in-the-bag IOL implantation and with modern sutureless techniques [19]. Previous studies of sutureless scleral fixation have reported mean deviations ranging from −0.5 to −1.2 diopters [19]. The slightly better refractive predictability in our series may be attributed to the standardized surgical technique and the predominant use of a single IOL model (Zeiss CT27SF, 91% of cases).

The positional stability data obtained from our detailed analysis (92.8% well centered; 93.7% without tilt) suggest that the proper surgical technique can achieve excellent anatomic outcomes. Our rates of significant tilt (4.5%) and decentration (7.2%) are lower than those reported in the recent literature. For comparison, studies of sutureless techniques have reported tilt rates of 8–12% and decentration rates of 5–15% [4]. This suggests that despite being technically more demanding, sutured fixation may provide superior long-term stability when executed with proper technique.

Our reoperation rate of a dislocated IOL due to suture breakage of 3.6% is notably lower than in previous reports, which have documented rates of 5–15% [19]. This difference may be attributed to several factors: our standardized surgical technique, the predominant use of a single IOL type, and the experience of our surgical team. However, it is important to note that our lower reoperation rate might also reflect a more conservative approach to managing minor IOL malposition.

The long-term complication profile in our series deserves careful consideration. The cumulative incidence of secondary glaucoma (16.8%) and retinal detachment (16.1%) over our follow-up period highlights the importance of regular monitoring. These rates are higher than those reported for primary cataract surgery but comparable to other studies of complex lens surgery in eyes with compromised capsular support (18). However, it is important to note that a definitive correlation between these long-term complications and the IOL suture fixation procedure cannot be conclusively established.

A significant consideration is that 33% of patients in our study required IOL suture fixation due to lens dislocation caused by PEX. Since PEX is both a major risk factor for lens instability and a known contributor to glaucoma development, it is likely that a substantial portion of the observed glaucoma cases can be attributed to the underlying PEX rather than the procedure itself. However, repeated surgical interventions, including IOL suture fixation, may exacerbate pre-existing PEX-related glaucoma and contribute to disease progression.

Additionally, as a large tertiary referral center frequently handling complex cases, a portion of the long-term complications could be attributed to the patients’ medical and surgical history, such as the above mentioned PEX or other underlying conditions, rather than the procedure itself. This context underscores the multifactorial nature of these outcomes and the need for cautious interpretation. These findings have several important clinical implications. First, they support suture fixation as a viable primary strategy for complex cases lacking capsular support rather than as a last resort. Second, they emphasize the importance of standardized surgical technique and IOL selection in achieving consistent outcomes. Third, they highlight the need for regular, long-term follow-up to monitor for late complications.

The study limitations include its retrospective nature, the single-center design, and the potential for selection bias inherent in a tertiary referral center population. Additionally, while the predominant use of a single IOL model and our standardized surgical approach enhances internal validity, it may limit generalizability. The variable follow-up duration, while allowing for long-term outcome assessment, may introduce survival bias in our complication analyses.

## 5. Conclusions

This extensive study of 332 eyes demonstrates that IOL (intraocular lens) suture fixation is both adequate and effective as a long-term solution for eyes lacking capsular support. The key findings include the following: There were favorable visual outcomes, with most patients maintaining or improving their vision. In cases where the best-corrected preoperative visual acuity was comparable to the postoperative result, patients still experienced an improved refractive outcome with reduced dependence on extensive refractive correction, such as contact lenses required for aphakic eyes. Success appears to depend on proper patient selection, a standardized surgical technique, and regular long-term follow-up. While this study has limitations due to its retrospective, single-center nature, the extended follow-up period of up to 17 years provides valuable insights into the procedure’s long-term efficacy.

## Figures and Tables

**Table 1:** Distribution of different IOL types with their respective target deviations of spherical equivalent in diopters.

IOL Model	CT27SF	AcriTec 27SF	MA60AC	Others
Number	272	31	11	18
Percentage	82%	9%	3%	5%
Spherical Equivalent Deviation	0.5	0.3	0.0	0.5
Range of Deviation	−0.86 to 1.69	−0.5 to 1.25	−0.06 to 1.1	−0.1 to 1.12

**Table 2:** Mean follow-up period of studies analyzing suture-fixated IOLs.

Authors	Study Period	Mean Follow-Up Period	Number of Eyes Included in Study
Kokame et al. [13]	1990–2012	72 months	118
Dimopopoulos et al. [17]	2004–2013	64 months	66
Wasiluk et al. [10]	2008–2012	64 months	29
Our study	2008–2022	30 months	332

## Data Availability

The data presented in this study are available upon request from the corresponding author due to privacy and ethical considerations.

## References

[1] Ascaso F.J., Huerva V., Grzybowski A. (2015). Epidemiology, Etiology, and Prevention of Late IOL-Capsular Bag Complex Dislocation: Review of the Literature. J. Ophthalmol..

[2] Subasi S., Yuksel N., Karabas V.L., Yilmaz Tugan B. (2019). Late in-the-bag spontaneous IOL dislocation: Risk factors and surgical outcomes. Int. J. Ophthalmol..

[3] Czajka M.P., Frajdenberg A., Stopa M., Pabin T., Johansson B., Jakobsson G. (2020). Sutureless intrascleral fixation using different three-piece posterior chamber intraocular lenses: A literature review of surgical techniques in cases of insufficient capsular support and a retrospective multicentre study. Acta Ophthalmol..

[4] Jacob S., Kumar D.A., Rao N.K. (2020). Scleral fixation of intraocular lenses. Curr. Opin. Ophthalmol..

[5] Gabor S.G.B., Pavlidis M.M. (2007). Sutureless intrascleral posterior chamber intraocular lens fixation. J. Cataract Refract. Surg..

[6] Agarwal A., Kumar D.A., Jacob S., Baid C., Agarwal A., Srinivasan S. (2008). Fibrin glue-assisted sutureless posterior chamber intraocular lens implantation in eyes with deficient posterior capsules. J. Cataract Refract. Surg..

[7] Prenner J.L., Feiner L., Wheatley H.M., Connors D. (2012). A novel approach for posterior chamber intraocular lens placement or rescue via a sutureless scleral fixation technique. Retina.

[8] Yamane S., Inoue M., Arakawa A., Kadonosono K. (2014). Sutureless 27-gauge needle-guided intrascleral intraocular lens implantation with lamellar scleral dissection. Ophthalmology.

[9] Krėpštė L., Kuzmienė L., Miliauskas A., Janulevičienė I. (2013). Possible predisposing factors for late intraocular lens dislocation after routine cataract surgery. Medicine.

[10] Wasiluk E., Krasnicki P., Dmuchowska D.A., Proniewska-Skrętek E., Mariak Z. (2019). The implantation of the scleral-fixated posterior chamber intraocular lens with 9/0 polypropylene sutures—Long-term visual outcomes and complications. Adv. Med. Sci..

[11] Althaus C., Sundmacher R., Pham D.T., Wollensak J., Rochels R., Hartmann C. (1994). Einnähung von Hinterkammerlinsen. Proceedings of the 8. Kongreß der Deutschsprachigen Gesellschaft für Intraokularlinsen Implantation.

[12] Price M.O., Price F.W., Werner L., Berlie C., Mamalis N. (2005). Late dislocation of scleral-sutured posterior chamber intraocular lenses. J. Cataract Refract. Surg..

[13] Kokame G.T., Yanagihara R.T., Shantha J.G., Kaneko K.N. (2018). Long-term Outcome of Pars Plana Vitrectomy and Sutured Scleral-Fixated Posterior Chamber Intraocular Lens Implantation or Repositioning. Am. J. Ophthalmol..

[14] Khan M.A., Gupta O.P., Smith R.G., Ayres B.D., Raber I.M., Bailey R.S., Hsu J., Spirn M.J. (2016). Scleral fixation of intraocular lenses using Gore-Tex suture: Clinical outcomes and safety profile. Br. J. Ophthalmol..

[15] Riedl J.C., Rings S., Schuster A.K., Vossmerbaeumer U. (2023). Intraocular lens dislocation: Manifestation, ocular and systemic risk factors. Int. Ophthalmol..

[16] Sinha R., Bansal M., Sharma N., Dada T., Tandon R., Titiyal J.S. (2017). Transscleral Suture-Fixated Versus Intrascleral Haptic-Fixated Intraocular Lens: A Comparative Study. Eye Contact Lens.

[17] Dimopoulos S., Dimopoulos V., Blumenstock G., Trevino-Rodriguez H., Bartz-Schmidt K.U., Spitzer M.S., Voykov  B. (2018). Long-term outcome of scleral-fixated posterior chamber intraocular lens implantation with the knotless Z-suture technique. J. Cataract Refract. Surg..

[18] Patel L.G., Starr M.R., Ammar M.J., Yonekawa Y. (2020). Scleral fixated secondary intraocular lenses: A review of recent literature. Curr. Opin. Ophthalmol..

[19] Sindal M.D., Nakhwa C.P., Sengupta S. (2016). Comparison of sutured versus sutureless scleral-fixated intraocular lenses. J. Cataract Refract. Surg..

